# Co-Activating Lattice Oxygen of TiO_2_-NT and SnO_2_ Nanoparticles on Superhydrophilic Graphite Felt for Boosting Electrocatalytic Oxidation of Glyphosate

**DOI:** 10.3390/nano14221824

**Published:** 2024-11-14

**Authors:** Wenyan He, Sheng Bai, Kaijie Ye, Siyan Xu, Yinuo Dan, Moli Chen, Kuo Fang

**Affiliations:** 1 College of Geology and Environment, Xi’an University of Science and Technology, Xi’an 710054, China; 22209226074@stu.xust.edu.cn (S.B.); 22209085036@stu.xust.edu.cn (K.Y.); 23209085041@stu.xust.edu.cn (S.X.); 24209215075@stu.xust.edu.cn (M.C.); 2 Shaanxi Provincial Key Laboratory of Geological Support for Coal Green Exploitation, Xi’an University of Science and Technology, Xi’an 710054, China; 3 College of Chemical Engineering, Beijing University of Chemical Technology, Beijing 100029, China; fangkuo@buct.edu.cn

**Keywords:** electrocatalytic oxidation, SnO_2_, glyphosate, •O_2_^−^ radicals

## Abstract

Glyphosate (GH) wastewater potentially poses hazards to human health and the aquatic environment, due to its persistence and toxicity. A highly superhydrophilic and stable graphite felt (GF)/polydopamine (PDA)/titanium dioxide nanotubes (TiO_2_-NT)/SnO_2_/Ru anode was fabricated and characterized for the degradation of glyphosate wastewater. Compared to control anodes, the GF/PDA/TiO_2_-NT/SnO_2_/Ru anode exhibited the highest removal efficiency (near to 100%) and a yield of phosphate ions of 76.51%, with the lowest energy consumption (0.088 Wh/L) for degrading 0.59 mM glyphosate (GH) at 7 mA/cm^2^ in 30 min. The exceptional activity of the anode may be attributed to the co-activation of lattice oxygen in TiO_2_-NT and SnO_2_ by coupled Ru, resulting in a significant amount of •O_2_^−^ and oxygen vacancies as active sites for glyphosate degradation. After electrolysis, small molecular acids and inorganic ions were obtained, with hydroxylation and dephosphorization as the main degradation pathways. Eight cycles of experiments confirmed that Ru doping prominently enhanced the stability of the GF/PDA/TiO_2_-NT/SnO_2_/Ru anode due to its high oxygenophilicity and electron-rich ability, which promoted the generation and utilization efficiency of active free radicals and defects-associated oxygen. Therefore, this study introduces an effective strategy for efficiently co-activating lattice oxygen in SnO_2_ and TiO_2_-NT on graphite felt to eliminate persistent organophosphorus pesticides.

## 1. Introduction

Over the past few years, extensive pesticides have been used to protect crops from pests and diseases, which adds up to approximately 9 million tons annually [1,2,3]. Among them, glyphosate, a representative and widely utilized organophosphate pesticide, constitutes about 15% of the global pesticide market [4,5,6,7]. Glyphosate wastewater poses significant environmental risks, as it may contaminate surface and groundwater through direct use, runoff, or leaching from the land application. Studies have documented its hepatotoxicity, immunotoxicity, endocrine toxicity, and reproductive toxicity effects on human and animal health [8,9]. Various methods, including electro-Fenton [4,10,11], ozone oxidation [12], photocatalysis [13,14], and electrochemical oxidation (ECO) [15,16,17,18,19] performed to eliminate or transform them into biodegradable substances. In particular, electrochemical oxidation, which relies on active free radicals, such as •OH, •O_2_^−^, and H_2_O_2_, is an excellent and less expensive method for degrading such persistent organic pollutants [3,20,21].

In the ECO process, the chemical and physical properties of the anode materials, including anode support, intermediate layer, and catalysts, play a critical role in determining its efficiency. High-performance anode support, such as Pt [22], Ni [23,24], boron-doped diamond (BDD) [25], Ti [26,27,28], and dimensionally stabilized anodes (DSAs) [29] were widely studied. Among them, titanium anode substrate has high tensile strength and corrosion resistance [30]. However, the passivation layer formed between the substrate and the electrolyte and electrode surfaces can decrease the stability of the electrodes, which hinders their practical application. Recently, superhydrophilic titanium dioxide nanotubes (TiO_2_-NT) [31,32] have effectively solved this problem, due to their unique three-dimensional structure [16]. Moreover, the presence of lattice oxygen and oxygen vacancies in TiO_2_-NT is crucial in enhancing their catalytic performance [33,34,35]. The new issue is that the conventional TiO_2_-NT grown on planar titanium plates or foils has a low space utilization [28,36,37], even if Ti networks are adopted [36]. In contrast, graphite felt (GF) has a high specific surface area, excellent electrical conductivity, and electrochemical stability [38]. Unfortunately, its hydrophobic surface properties resulted in a large mass transfer barrier for the diffusion of water and pollutants into the catalyst sites [39], preventing the emission of bubbles from the electrode surface and generating active radicals [40], thus reducing the degradation efficiency of hydrophilic glyphosate. Therefore, the combination of TiO_2_-NT and GF could handle the above problems of electrode hydrophobicity and provide a large surface area for catalyst loading. However, how to make TiO_2_-NT stably load on GF and activate lattice oxygen has become a new question.

To further enhance the surface area of the electrode substrates, improve catalyst dispersion and stability, and facilitate efficient electron transfer, extensive research has been conducted on electrode intermediate layers, including metallic oxide, polymer and metal-organic frameworks (MOFs), and so on. Among them, polydopamine (PDA), produced by the self-polymerization of dopamine (DA), exhibits strong adhesion on various material surfaces due to multiple covalent and non-covalent interactions of the catechol and amine groups [41]. Thus, the problem of loading TiO_2_-NTs on graphite felt can be effectively solved using PDA. Besides, these hydrophilic groups can improve the hydrophilicity of the electrode surface, thus enhancing the electrode-solution mass transfer efficiency and reducing the resistance during electron transfer [26,42,43]. Moreover, PDA exhibits excellent electrical conductivity and electron-rich properties, enhancing the overall electron transfer efficiency of the electrode.

The catalyst plays a decisive role in the efficient degradation of organic matter via active radicals. Compared to PbO_2_ and TiO_2_, SnO_2_ is known for its oxygen vacancies, highly reactive lattice oxygen and acidic catalytic properties [17,44]. However, pure SnO_2_ cannot be directly used due to its low conductivity [45,46,47,48]. Besides, how to enhance the metal–oxygen (M-O) covalency in SnO_2_ is the trigger for the lattice oxygen-mediated mechanism, which is an excellent alternative pathway to overcoming the larger reaction energy barrier [49]. The greater hybridization degree between the d-orbit of M and the p-orbit of O represents that the lattice oxygen is activated and participates in O_2_ production. Thus, Ti, Co, F, B, and Ru are commonly doped to improve the stability and catalytic activity of electrodes [15,36,50]. For example, 95.6% degradation efficiency and 61.39% defluorination rate of chlorinated polyfluorinated ether sulfonate (F-53B) were obtained by F-doped SnO_2_ electrode [17]. Yang et al. [51] prepared a SnO_2_/(CoTeO_3_)_2_ heterojunction catalyst for electrocatalytic oxidative degradation of glucose, which also possessed excellent H_2_ production capability. In these cases, SnO_2_ could form adsorbed free radicals through reactive lattice oxygen atoms during the electrocatalytic oxidation, thus showing excellent catalytic performance for pollutant removal [52]. To further enhance electron transfer efficiency, Ma et al. [53] designed a novel CuFe-Sb-SnO_2_ anode for efficient degradation of ciprofloxacin, and the Cu and Fe in the electrode led to surface reconstruction, which reduced charge transfer resistance between Sn and Sb, thereby promoting in situ •OH production. Besides, Ti/La/Co-Sb-SnO_2_ electrodes doped with La as the active layer and Co as the intermediate layer were synthesized to prolong their lifespan without compromising their ECO activity [54]. Moreover, MOF magnetic particles Fe_3_O_4_@UiO-66-NH_2_@PANI were adsorbed onto the Ti/Sb-SnO_2_ electrodes surface to construct an electrooxidation dynamic system, enhancing the production of hydroxyl radicals and reducing the toxicity of methotrexate [55]. However, increasing the number of oxygen vacancies and active free radicals is an attractive issue. Research confirmed that the doping of Ru could change the electronic structure of the electrode and promote electron transfer, making it easier for lattice oxygen to participate in the reaction [56]. Moreover, Ru doping can introduce more oxygen vacancies, which serve as active sites and promote lattice oxygen activation. Wang et al. [57] found that the presence of Ru in TiO_2_ could make the Ti sites substitute with Ru periodically arranged due to the similarity in the crystal structures of rutile RuO_2_ and TiO_2_, which helps to obtain electrochemically and structurally stable catalysts. Ren et al. [40] reported a stable Sb-doped Ti/RuO_2_-IrO_2_-SnO_2_ anode for the degradation of aniline using diverse power supply modes. The pulse mode was more efficient than the direct current supply mode, due to the synergism of •OH, •OCl and ^1^O_2_, and Sb doping facilitated electron transport, enlarging the electrochemical active surface area, enhancing stability, and increasing electrocatalytic activity. Additionally, as a typical tetragonal structure of the substances, RuO_2_, TiO_2_, and SnO_2_ have similar chemical bonding modes but different conduction and valence band positions, thus the effect of Ru doping on lattice oxygen co-activation in SnO_2_ and TiO_2_ needs to be studied.

Therefore, this project proposed to prepare an efficient GF/PDA/TiO_2_-NT/SnO_2_/Ru composite anode for the electrocatalytic oxidation of glyphosate wastewater, comprehensively assessed the overall performance of the electrode by examining its structure, morphology, hydrophilicity, electrochemical characterization test, and electrode stability and life-extension experiment. A possible degradation mechanism of glyphosate was proposed by radical quenching experiments and electron spin resonance spectroscopy (EPR) tests. The intermediates, by-products, and dephosphorization efficiency of glyphosate were verified by LC-MS and spectrophotometry, respectively. This study aims to provide a simple and highly efficient method for organophosphorus wastewater degradation.

## 2. Materials and Methods

### 2.1. Chemicals and Materials

Glyphosate, titanium oxide, trimethylenediamine, sodium nitrite, 3-hydroxytyramine hydrochloride, sodium phosphite dibasic pentahydrate, glyphosine, and terephthalic acid were obtained from MACKLIN^®^ (Sydney, Australia). Ethanol, potassium bromide, and sodium chloride were bought from Kermel^®^. N, N-Dimethylformamide (DMF), and propanediol were purchased from Tianjin Fuyu Chemical^®^ (Tianjin, China). Tin (Sn(IV)) chloride pentahydrate (SnCl_4_·5H_2_O) was obtained from Shanghai Zhanyun Chemical Co., Ltd. (Shanghai, China). DNA-grade Tris-HCI solution was obtained from RHAWN^®^. RuCl_3_ was purchased from Shanghai Haohong Biopharmaceutical Co., Ltd. (Shanghai, China).

### 2.2. Preparation of GF/PDA/TiO_2_-NT/SnO_2_/Ru Electrode

Preparation of GF/PDA/TiO_2_-NT/SnO_2_ electrode. First, the graphite felt (GF, cylinder dimensions: φ 1 cm × 2 cm) was cleaned with anhydrous ethanol and deionized water in ultrasonic waves to remove surface impurities and then dried in an oven at 333 K for 8 h. The pre-treated GF was immersed in 13.05 mM dopamine solution and ultrasonicated at room temperature for 8 h to obtain PDA-modified GF [31]. Then 0.8 g TiO_2_ nanotubes, prepared according to the literature [58,59], were put into the solution and continued the ultrasound for 12 h [26,42]. The obtained GF/PDA/TiO_2_-NT was placed in 100 mL propanediol containing 20 g of SnCl_4_·5H_2_O at room temperature, then it was annealed in an oven at 373 K for 30 min and a muffle furnace at 773 K for 20 min to form SnO_2_ and the anatase phase of TiO_2_ [60,61]. The above process was repeated eight times to obtain GF/PDA/TiO_2_-NT/SnO_2_ electrodes.

**Figure 1:**
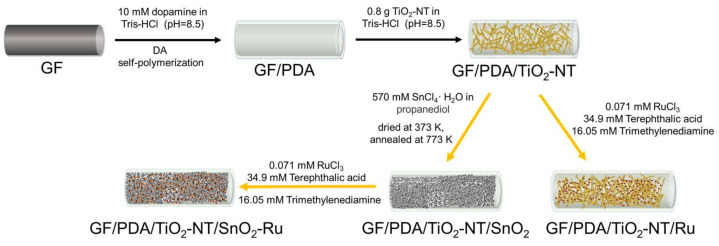
Schematic diagram for preparation different electrodes.

Preparation of GF/PDA/TiO_2_-NT/SnO_2_/Ru electrode (Figure 1). 0.175 g trimethylenediamine and 0.055 g terephthalic acid were dissolved in 30 mL N, N-dimethylformamide under sonication, added 2 mL, 20 mM RuCl_3_ aqueous solution and stirred at room temperature for 10 min. The mixed solution was transferred to a 50 mL Teflon-lined stainless autoclave and put in prepared GF/PDA/TiO_2_-NT/SnO_2_. The autoclave was placed in an oven and heated at 403 K for 24 h [57,62,63]. After naturally cooling to room temperature, the electrode was washed several times with ethanol and dried at 333 K for 5 h. For comparison, the electrodes of GF/PDA/TiO_2_-NT and GF/PDA/TiO_2_-NT/Ru were prepared according to the above procedure.

### 2.3. Analysis Methods

Scanning electron microscopy (SEM, JEM-2100F, Tokyo, Japan) and energy-dispersive spectroscopy (EDS, JEM-2100F, Maebashi, Japan) were used to analyze the morphology and elemental composition of the prepared electrodes. The valence states of the electrode surface elements were tested by an X-ray photoelectron spectrometer (XPS, Thermo Fisher Scientific ESCALAB 250 XI, Waltham, MA, USA). The crystal structure of the electrodes was investigated by a D8 powder X-ray diffractometer (XRD, Bruker, Billerica, MA, USA). The concentrations of the tin and ruthenium loaded on the GF/PDA/TiO_2_-NT/SnO_2_/Ru anode and leached in the electrolyte after electrolysis were measured with an inductively coupled plasma optical emission spectrometer (ICP-OES 720ES, Agilent, Santa Clara, CA, USA). The intensity of •OH and •O_2_^−^ of the electrodes were examined by electron spin resonance spectroscopy (EPR, Bruker A300, Ettlingen, Germany). An electrochemical working station (CHI 660E, Chenhua Instruments, Shanghai, China) was used to investigate the cyclic voltammetry (CV) and linear sweep voltammetry (LSV). Electrochemical impedance spectroscopy was measured in the frequency range of 100 kHz to 10 MHz at open circuit potential with a signal amplitude of 10 mV [64]. The chemical oxygen demand (COD) concentration was measured by a COD rapid analyzer (Lianhua Technology, Shanghai, China).

### 2.4. Electrochemical Oxidation Experiments

The electrocatalytic oxidation of glyphosate wastewater was conducted using a conventional three-electrode system. A 50 mL H-type cell equipped with a proton exchange membrane was employed. The working electrode comprised a GF/PDA/TiO_2_-NT/SnO_2_/Ru composite with varying Ru loadings, while a mercury sulfate electrode (MSE) and a platinum electrode served as the reference and counter electrodes, respectively. Glyphosate solutions, ranging from 0.059 to 1.18 mM, were electrolyzed in 0.5 mol/L NaCl under varying pH conditions (3–7) and current densities (3–10 mA/cm^2^). Samples of 2 mL were collected every ten minutes. Glyphosate concentrations were quantified via UV spectrophotometry at 242 nm. Additionally, PO₄^3−^ concentrations were determined using ammonium molybdate spectrophotometry at 710 nm. Ammonium concentration was measured via Nessler’s reagent spectrophotometric method. NO_3_^−^ concentration was tested by UV spectrophotometry at 220 nm and 275 nm. The electrolysis efficiency of glyphosate degradation was calculated according to Equation (1).

(1) $$\mathrm{Electrolysis}\;\mathrm{efficiency}=1-\left(\frac{C_{t}}{C_{0}}\times 100\%\right)$$

where C_0_ and C_t_ were the glyphosate concentration (mM) at initial and time t (min). respectively.

The energy consumption for electrochemical degradation of glyphosate wastewater was calculated according to the electric energy per order (E_EO_) (Wh/L).

(2) $$E_{\mathrm{EO}}=\frac{\mathrm{UIt}}{\mathrm{Vlog}\left(\frac{C_{0}}{C_{t}}\right)}$$

where U was the applied potential (V), I was the current (A), V was the solution volume (L), t was the electrolysis time, C_0_ and C_t_ were the pollutant concentration (mM) at initial and time t (h), respectively.

The total organic carbon (TOC) was measured using the Water Detective 3-Multi-Parameter Spectral Water Quality Tester. The TOC removal rate was calculated according to Equation (3).

(3) $$\mathrm{TOC}\;\mathrm{removal}\;\mathrm{rate}=\frac{C_{t}}{C_{0}}\times 100\%$$

where C_0_ and C_t_ were the TOC concentration (mM) of glyphosate wastewater at initial and time t (min), respectively.

## 3. Results

### 3.1. Electrodes Characterization

**Figure 2:**
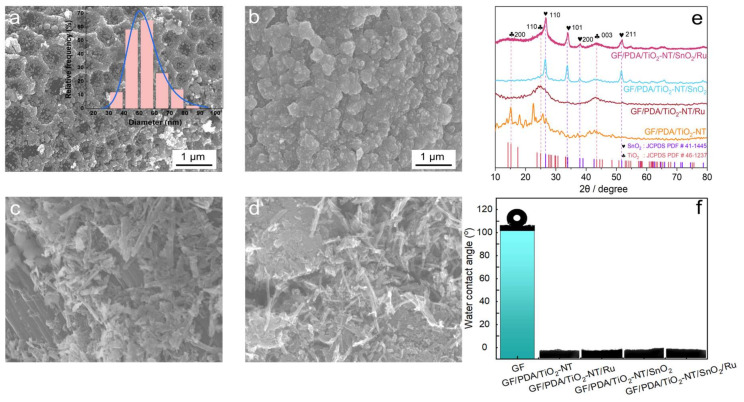
SEM images of (**a**) GF/PDA/TiO_2_-NT/SnO_2_/Ru, (**b**) GF/PDA/TiO_2_-NT/SnO_2_, (**c**) GF/PDA/TiO_2_-NT/Ru, (**d**) GF/PDA/TiO_2_-NT. (**e**) XRD patterns and (**f**) water contact angle of the four electrodes.

SEM images of the GF/PDA/TiO_2_-NT/SnO_2_/Ru anode exhibited homogeneous honeycomb structures (Figure 2a), predominantly composed of SnO_2_ nanoparticles with an average size of 43.83 nm. In the absence of Ru, the SnO_2_ on the GF/PDA/TiO_2_-NT/SnO_2_ presented an agglomerated morphology (Figure 2b). This was probably due to Ru loading altering the crystal growth dynamics and optimizing the surface energy, and reducing the mutual attraction of SnO_2_ nanoparticles. Figure 2c illustrates that without SnO_2_, TiO_2_ nanofibers were observed on the surface of the GF/PDA/TiO_2_-NT/Ru electrode, with only slight changes compared to the surface of GF/PDA/TiO_2_-NT (Figure 2d), indicating that Ru doping did not significantly affect the morphology of TiO_2_-NT.

The XRD patterns in Figure 2e revealed peaks at 26.54°, 33.86°, and 37.95°, as well as 51.78° for both the GF/PDA/TiO_2_-NT/SnO_2_/Ru and GF/PDA/TiO_2_-NT/SnO_2_ electrodes, corresponding to the (110), (101), (200), and (211) planes of the SnO_2_ crystal (JCPDS 41-1445) [2]. In particular, the lattice oxygen on the SnO_2_ (110) surface played a vital role in the oxidation process [65]. Compared to the GF/PDA/TiO_2_-NT/SnO_2_ electrode, there was a decrease in diffraction peaks of SnO_2_ with Ru doping, likely due to alterations in the crystal structure of the SnO_2_. The peaks at 15.20°, 24.93°, and 43.51° on all four electrodes were assigned to the (200), (110), and (003) facets of the TiO_2_ crystal (JCPDS 46-1237), respectively. You et al. [35] verified that the surface of TiO_2_ (111) could offer oxygen vacancies for water molecule dissociation to form two hydroxyl groups, which may further generate hydroxyl radicals (•OH). No Ru diffraction peak was observed due to its low content, while ICP and XPS analysis confirmed its existence. Compared to the highly hydrophobic GF (with a water contact angle of 101.8°), the electrodes showed superhydrophilicity after modification by PDA and TiO_2_-NT (Figure 2f), which effectively enhanced the mass transfer efficiency of pollutants to the electrode surface. There was no effect on the hydrophilicity of the electrode after doping SnO_2_ and Ru.

**Figure 3:**
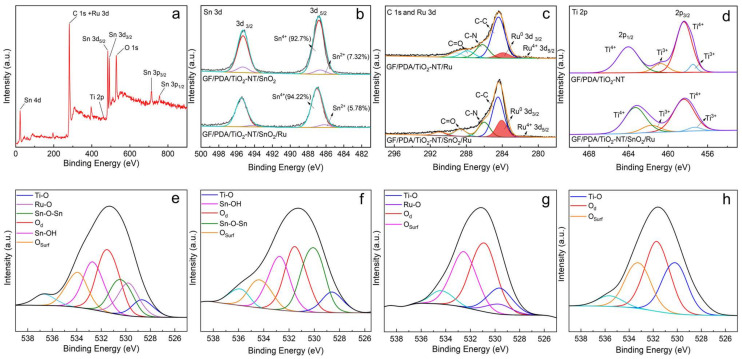
(**a**) A full-scale XPS spectrum of GF/PDA/TiO_2_-NT/SnO_2_/Ru. XPS spectra of (**b**) Sn 3d, (**c**) C 1s and Ru 3d, (**d**) Ti 2p and O 1s of (**e**) GF/PDA/TiO_2_-NT/SnO_2_/Ru, (**f**) GF/PDA/TiO_2_-NT/SnO_2_, (**g**) GF/PDA/TiO_2_-NT/Ru, (**h**) GF/PDA/TiO_2_-NT.

In Figure 3a, the existence of C, O, Ti, Sn, and Ru elements in the GF/PDA/TiO_2_-NT/SnO_2_/Ru electrode can be distinctly seen in the full-scale spectrum of XPS. For the GF/PDA/TiO_2_-NT/SnO_2_/Ru and GF/PDA/TiO_2_-NT/SnO_2_ anodes, the Sn 3d XPS spectra showed a distinct spin-orbit splitting, with the 3d_5/2_ and 3d_3/2_ peaks at 486.8 eV and 495.3 eV, respectively, indicating that Sn mainly presented in the Sn^4+^ valance state in the catalytic layer (Figure 3b), as the ratio of Sn^2+^ was less than 10%. The intensity of the Sn 3d peak decreased after Ru doping, indicating that the chemical environment of Sn became less ordered. The Ru incorporation may introduce lattice defects that destroy the ordering of the SnO_2_ lattice, confirmed by XRD data showing broader peaks in Figure 2e. Besides, the chemical bond between Sn and the oxygen atom was affected by Ru incorporation, which showed a positive shift in Sn 3d peak position compared to that of GF/PDA/TiO_2_-NT/SnO_2_, demonstrating that the chemical environment of the surface Sn species was altered due to the strong chemical interaction between SnO_2_ and Ru [66]. Although the XPS spectra of Ru 3d and C 1s partially overlapped, it was still possible to recognize the signals of Ru 3d_5/2_ and Ru 3d_3/2_ (Figure 3c). The predominance of the Ru 3d_3/2_ peak indicated the presence of metallic Ru. In contrast, the Ru 3d_5/2_ peak represented Ru^4+^, which could take the place of Sn^4+^ in SnO_2_ to form the Ru-O bond, increasing the binding energy of SnO_2_ compared to GF/PDA/TiO_2_-NT/SnO_2_, whether the SnO_2_ existence did not affect the valence state of Ru. The three diffraction peaks of C 1s from GF were assigned to C-C (284.8 eV), C-N (286.1 eV), and C=O (288.6 eV) bonds, respectively, and existed in both electrodes. Ti 2p XPS spectra in Figure 3d showed that the core energy levels of Ti^4+^ 2p_3/2_ and Ti^4+^ 2p_1/2_ on GF/PDA/TiO_2_-NT were located at 458.4 eV and 464.0 eV, respectively. The binding energy of Ti^4+^ 2p_1/2_ on GF/PDA/TiO_2_-NT/SnO_2_/Ru shifted to a lower value, indicating that Ru doping may result in the transfer of electrons to Ti, thereby changing the electronic environment of Ti. The presence of Ti^3+^ verified the formation of oxygen vacancies, which were also observed in the GF/PDA/TiO_2_-NT/SnO_2_/Ru. From the XPS spectra of O 1s (Figure 3e–h), three peaks at 530.4 eV, 531.4 eV, and 534.0 eV can be ascribed to the lattice oxygen (O_L_), defects-associated oxygen (O_d_), and surface-adsorbed oxygen of metal oxides (O_surf_), respectively [67]. Besides, the O_L_ can be de-convoluted into Sn-O-Sn (530.37 eV), Ru-O (529.8 eV), and Ti-O (529.5 eV), and the peak at 532. 63 eV may be attributed to hydroxide species of Sn-OH [57,68,69]. For GF/PDA/TiO_2_-NT/SnO_2_/Ru, the increase in the ratio of defects-associated oxygen and decrease of the content and binding energy of Sn-O-Sn and the formation of Ru-O, suggesting that the splintering of TiO_2_-NT and SnO_2_ crystals after Ru incorporation generated lattice defects (Figure 3e–f). This was consistent with the increased Ti^3+^ content, as shown in Figure 3d, to maintain the overall charge neutrality. Besides, the contents of defect-associated oxygen species of GF/PDA/TiO_2_-NT/Ru increased compared to that of GF/PDA/TiO_2_-NT (Figure 3g–h), implying the significant changes in metal–O bonding with Ru doping. Therefore, the co-activation mechanism of lattice oxygen in TiO_2_-NT and SnO_2_ by Ru was as follows: first, Ru acted as an electron donor, which could facilitate electron transfer to the lattice oxygen and enhance its reactivity; second, the incorporation of Ru introduced oxygen vacancies, which lowered the binding energy of the lattice oxygen and created active sites for the electrocatalytic oxidation of glyphosate—additionally, the formation of Ru-O bonds provided further active sites that enhanced the reactivity of the lattice oxygen; third, Ru doping increased the electrochemical active area and altered the surface chemistry of TiO₂-NT and SnO₂, exposing more lattice oxygen atoms, thus increasing the electrocatalytic oxidation efficiency of glyphosate.

### 3.2. Optimization of Glyphosate Degradation Conditions

**Figure 4:**
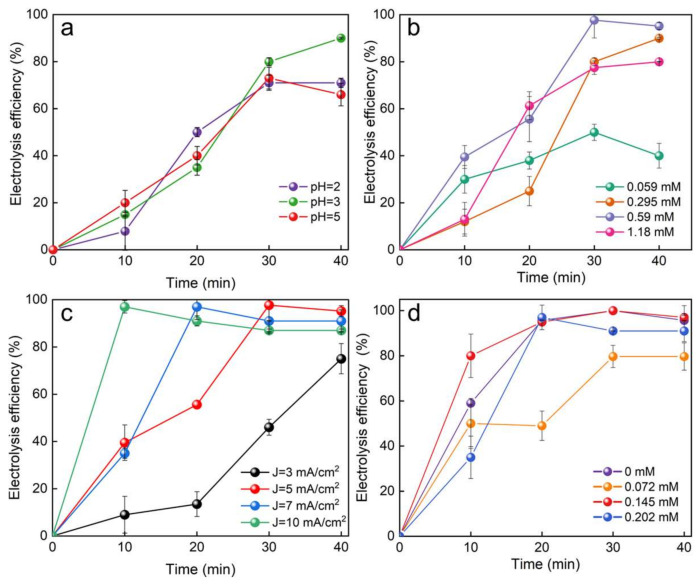
Effects of (**a**) pH, (**b**) initial concentration of glyphosate, (**c**) current density, (**d**) Ru loading on the glyphosate degradation efficiency of GF/PDA/TiO_2_-NT/SnO_2_/Ru electrode.

Several vital parameters, including initial pH, current density, initial glyphosate concentration, and Ru loading on GF/PDA/TiO_2_-NT/SnO_2_/Ru, were investigated for their impact on the degradation efficiency of glyphosate. Compared to pH = 2 and 5, the highest electrocatalytic oxidation efficiency of glyphosate (91%) on GF/PDA/TiO_2_-NT/SnO₂/Ru electrode was achieved at pH = 3 and a current density of 5 mA/cm^2^ with an initial concentration of 0.295 mM (Figure 4a). At pH = 3, the anode surface became positively charged, enhancing the adsorption of negatively charged glyphosate molecules through electrostatic attraction. Additionally, large amounts of H^+^ enhanced the oxygen evolution overpotential and prevented side reactions, such as oxygen evolution and decomposition of •OH and •O^2−^ [2]. At pH = 2, competing reactions, such as hydrogen-gas generation may have interfered with the production of •OH. The degradation efficiency of glyphosate on the GF/PDA/TiO_2_-NT/SnO_2_/Ru anode under alkaline conditions was not thoroughly investigated because the stronger oxygen evolution reaction reduced the availability of •OH, mainly due to the decreased oxidation potential of •OH from +2.85 V vs. SHE in acidic conditions to +2.02 V vs. SHE in alkaline conditions [70].

In 95% of the countries surveyed, glyphosate may pose a moderate to high risk, with a concentration of 0.62 mM [71]. Therefore, the degradation efficiency of glyphosate within the concentration range of 0.059–1.18 mM was studied on the GF/PDA/TiO_2_-NT/SnO_2_/Ru electrode. The degradation efficiency of glyphosate was further increased by about 50% when the initial concentration increased from 0.059 mM to 0.59 mM (Figure 4b). As illustrated in Figure 4c, at a current density of 10 mA/cm^2^, 97% degradation efficiency of glyphosate was obtained within 10 min, whereas it took 20 min and 30 min for a current density of 7 mA/cm^2^ and 5 mA/cm^2^, respectively. This phenomenon can be attributed to the enhanced electron transfer rate and the production of reactive oxygen species at higher current densities. However, the too-high current density may lead to oxygen evolution and conversion of •OH to H_2_O_2_ and HO_2_• [72], which compete with glyphosate degradation at the anode surface, resulting in greater energy consumption (Table S1).

The impact of Ru loading on the GF/PDA/TiO_2_-NT/SnO_2_/Ru electrode for the degradation efficiency of glyphosate was investigated (Figure 4d). In comparison to the absence of Ru, the introduction of Ru (0.072–0.145 mM) rapidly improved the degradation efficiency of glyphosate within 10 min. This enhancement can be attributed to the activation of the lattice oxygen in SnO_2_ and TiO_2_-NT by Ru to generate active free radicals and oxygen vacancies, promoting the adsorption and degradation of glyphosate. However, excessive Ru loading leads to over-oxidation, resulting in catalyst deactivation due to the formation of inactive Ru oxides. Therefore, the optimized conditions were pH = 3, glyphosate concentration was 0.59 mM at 7 mA/cm^2^, and the Ru loading was 0.145 mM on the GF/PDA/TiO_2_-NT/SnO_2_/Ru.

### 3.3. Synergistic Degradation of Glyphosate

**Figure 5:**
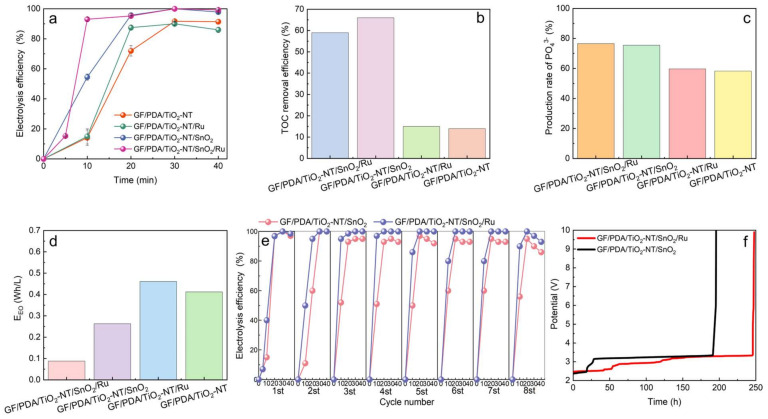
(**a**) Degradation efficiency, (**b**) TOC removal rate, (**c**) production rate of PO_4_^3−^, (**d**) energy consumption on GF/PDA/TiO_2_-NT, GF/PDA/TiO_2_-NT/Ru, GF/PDA/TiO_2_-NT/SnO_2_, GF/PDA/TiO_2_-NT/SnO_2_/Ru electrodes. (**e**) Recycle experiments of glyphosate degradation, (**f**) accelerated lifetime test of GF/PDA/TiO_2_-NT/SnO_2_, GF/PDA/TiO_2_-NT/SnO_2_/Ru electrodes.

Control experiments were performed to evaluate the electrocatalytic properties of GF/PDA/TiO_2_-NT/SnO_2_/Ru for the degradation of glyphosate. Figure 5a indicated that the degradation efficiency of glyphosate at the GF/PDA/TiO_2_-NT/SnO_2_/Ru electrode was close to 100%, with Sn and Ru loading densities of 0.126 mg/cm^2^ and 0.015 mg/cm^2^, respectively, comparable to the performance of the GF/PDA/TiO_2_-NT/SnO_2_ electrode in 30 min. Notably, Ru doping in GF/PDA/TiO_2_-NT/SnO_2_/Ru significantly improved the degradation rate of glyphosate by 40% in 10 min. This was because Ru doping co-activated the lattice oxygen of SnO_2_ and TiO_2_ and generated more defects-associated oxygen (Figure 3e-h), which could be used as active sites to promote the adsorption and activation of oxygen molecules and promote the generation of •OH and •O_2_^−^ for glyphosate degradation. Compared to GF/PDA/TiO_2_-NT, the degradation efficiency of glyphosate on GF/PDA/TiO_2_-NT/Ru was increased by 15% in 20 min, indicating Ru also had an activation effect on TiO_2_-NT alone. Besides, the GF/TiO_2_-NT/SnO_2_/Ru electrode achieved an approximate 89% removal rate of glyphosate (Figure S1), with an 11% efficiency loss compared to that of the GF/PDA/TiO_2_-NT/SnO_2_/Ru electrode, possibly due to the diminished stability and dispersibility of the catalyst on GF without PDA. Additionally, the effect of superhydrophilic GF/PDA/TiO_2_-NT support on the degradation of glyphosate should not be underestimated, as in the absence of Ru, TiO_2_ nanotubes can also generate reactive oxygen species for the degradation of glyphosate.

The TOC mineralization efficiency of glyphosate on the GF/PDA/TiO_2_-NT/SnO_2_/Ru electrode was approximately 59% (Figure 5b), indicating the incomplete mineralization process. The presence of intermediate products, such as oxalic acid, formic acid, and acetic acid obtained after glyphosate degradation, was confirmed by LC-MS analysis (Figure S2). The most exciting finding was that these newly formed acids could be further reduced to alcohols, which can serve as energy sources through a straightforward reduction reaction. The yield of phosphate ions in the degradation products was 76.51%, which confirmed the dephosphorization reaction (Figure 5c). The GF/PDA/TiO_2_-NT/SnO_2_ electrode exhibited the highest TOC removal rate of 65%, suggesting that these acids may be further oxidized to CO_2_, which was not conducive to energy utilization. As illustrated in Figure 5d, the energy consumption of the GF/PDA/TiO_2_-NT/SnO_2_/Ru electrode was approximately one-third that of the GF/PDA/TiO_2_-NT/SnO_2_ electrode, suggesting that the incorporation of Ru accelerated charge transfer reactions between TiO_2_, SnO_2_ and Ru, thereby facilitating the generation of active radical. The increased charge transfer efficiency likely contributed to the reduction of energy consumption and the overall enhancement of electrocatalytic performance, indicating the beneficial role of Ru in the composite material.

To further evaluate the practical application potential of GF/PDA/TiO_2_-NT/SnO_2_/Ru electrode, over eight consecutive electrocatalytic oxidation cycles of glyphosate were performed under optimal conditions (Figure 5e). Notably, the GF/PDA/TiO_2_-NT/SnO_2_/Ru electrode sustained a degradation efficiency of 93% by the eighth cycle, while 86% for GF/PDA/TiO_2_-NT/SnO_2_, underscoring its exceptional stability. The ICP analysis illustrated that the concentration of Sn and Ru in the eighth degraded solution was 5.3 × 10^−5^ mM and 5.8 × 10^−4^ mM, respectively, indicating that almost negligible catalyst dissolution from the electrode occurred. Besides, stability testing of both GF/PDA/TiO_2_-NT/SnO_2_ and GF/PDA/TiO_2_-NT/SnO_2_/Ru anodes was carried out. Compared to the former electrode, the accelerated service life of the latter electrode was 245 h (Figure 5f), with a slight increase in potential, further confirming the high stability of the GF/PDA/TiO_2_-NT/SnO_2_/Ru electrode due to high chemical stability provided by Ru doping. Besides, the simulated glyphosate wastewater experiments confirmed that compared with glyphosine or phosphite alone, the degradation efficiency of glyphosate was significantly affected when the two co-existed due to their competition for both active sites and active free radicals simultaneously (Figure S3a). Thus, the high current density of 20 mA/cm^2^ was applied to boost the generation of active radicals, 100% degradation efficiency of glyphosate was obtained in 100 min, and the COD removal efficiency was increased by about 30% compared to at 7 mA/cm^2^ (Figure S3b). This result indicated the potential application of GF/PDA/TiO_2_-NT/SnO_2_/Ru electrodes in organophosphorus wastewater treatment.

**Figure 6:**
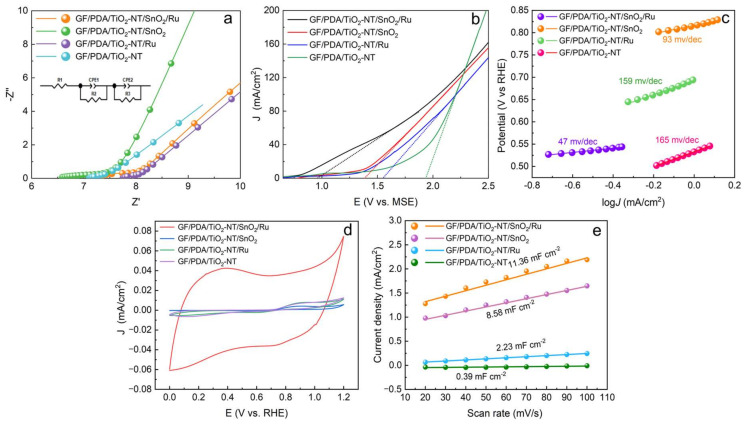
Electrochemical characterization of the four electrodes: (**a**) EIS curves, (**b**) LSV curves, (**c**) Tafel plots, (**d**) CV, (**e**) C_dl_ of GF/PDA/TiO_2_-NT/SnO_2_, GF/PDA/TiO_2_-NT/SnO_2_/Ru electrodes.

As illustrated in Figure 6a, the difference in R_s_ of the four electrodes was probably due to the difference in the solution impedance in the electrode channel, as the morphology of the four electrodes’ surfaces changed obviously, which can be inferred that the surface area and pore structure of the electrode have changed correspondingly, thus affecting the current flow at the electrode–electrolyte interface and making the R_s_ values slightly different. Compared to the other electrodes, the oxygen evolution potential (OEP) for the GF/PDA/TiO_2_-NT/SnO_2_/Ru anode was relatively low, at 0.96 V vs. RHE; without Ru and SnO_2_, it increased to 1.99 V vs. RHE for GF/PDA/TiO_2_-NT anode (Figure 6b). Due to the presence of Cl^−^, the faradic current was related to the mixed Cl_2_ and O_2_ evolutions. As a comparison, the O_2_ evolution directly occurred without observing the flat curve at the beginning of the curve without Cl^−^ (Figure S4). The OEP of the GF/PDA/TiO_2_-NT/SnO_2_/Ru electrode was approximately 0.13 eV lower than that in the absence of Cl^−^, indicating the addition of Cl^⁻^ ion enabled O_2_ evolution to occur at a lower potential. Figure 6c indicates that the GF/PDA/TiO_2_-NT/SnO_2_/Ru anode has the smallest Tafel slope (47 mV/dec) compared to the other three electrodes, indicating the minimal overpotential. Furthermore, a noteworthy change in current density was observed on the GF/PDA/TiO_2_-NT/SnO_2_/Ru anode in the potential range (Figure 6d), resulting in the largest double-layer capacitance (C_dl_) (11.36 mF/cm^2^), which was 1.32 times and 5.09 times that of the GF/PDA/TiO_2_-NT/SnO_2_ and GF/PDA/TiO_2_-NT/Ru (Figure 6e), respectively. Given the linear relationship between the electrochemically active surface area (ECSA) and the double-layer capacitance, a larger C_dl_ indicates a larger ECSA [73]. Consequently, the GF/PDA/TiO₂-NT/SnO₂/Ru electrode demonstrated a significantly larger active surface area and vaster active sites than the other three electrodes. Therefore, it could provide more sites for direct electrochemical oxidation, facilitate the capture of organic pollutants, and promote the generation of active free radicals under the same applied potential [2], thereby enhancing the degradation efficiency of glyphosate. Table 1 showed that more electrolysis time (120–360 min) and a much higher current density of 10–50 mA/cm^2^ was required for glyphosate degradation on the reported anodes, thus requiring more energy consumption. This indicated the excellent performance of the GF/PDA/TiO_2_-NT/SnO_2_/Ru anode with the lowest energy consumption of 0.088 Wh/L.

### 3.4. Proposed Mechanism for Glyphosate Degradation

**Figure 7:**
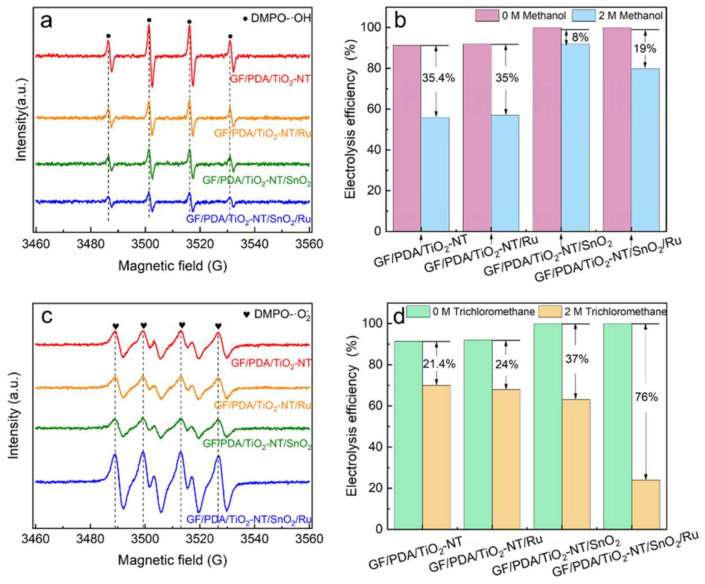
(**a**) EPR tests for •OH and (**c**) •O_2_^−^ on different electrode; (**b**) •OH quenching experiments and (**d**) •O_2_^−^ quenching experiments on different electrodes.

To elucidate the contribution of indirect oxidation in the electrocatalytic degradation of glyphosate, the EPR experiment with 5,5-dimethyl-1-pyrroline N-oxide (DMPO) as the trapping agent was performed. The presence of four characteristic peaks of DMPO-•OH (intensity ratio of 1:2:2:1) was observed on all the electrodes (Figure 7a). The GF/PDA/TiO_2_-NT exhibited the highest •OH generation capacity, and it gradually decreased with Ru and SnO_2_ doping, suggesting that the ability to generate •OH was gradually weakened. Hydroxyl radical quenching experiments confirmed that the degradation efficiency of glyphosate was only reduced by 19% on the GF/PDA/TiO_2_-NT/SnO_2_/Ru anode as 2 M methanol was added as a quenching scavenger (Figure 7b). The results suggested that the high degradation efficiency of glyphosate was not predominantly driven by •OH radicals. The EPR experiment with DMPO/DMSO as a trapping agent verified the presence of DMPO-•O_2_^−^ (four characteristic peak intensity ratio of 1:1:1:1), and the GF/PDA/TiO_2_-NT/SnO_2_/Ru electrode exhibited the highest intensity of •O_2_^−^ compared to the other electrodes (Figure 7c). This was because the Ru doping may enhance the activity of lattice oxygen in SnO_2_ and TiO_2_, promoting the generation of •O_2_^−^. Figure 7d exhibited that when 2 M trichloromethane was added, a 76% decrease in the degradation efficiency of glyphosate on the GF/PDA/TiO_2_-NT/SnO_2_/Ru electrode was obtained, indicating that the degradation process was primarily dominated by an indirect oxidation mechanism involving •O_2_^−^.

**Figure 8:**
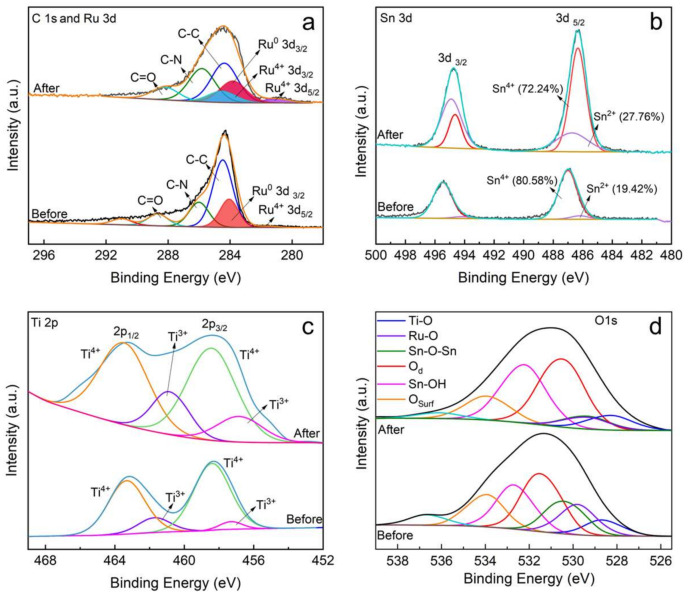
Comparison of XPS spectra of (**a**) C 1s and Ru 3d, (**b**) Sn 3d; (**c**) Ti 2p, (**d**) O 1s of GF/PDA/TiO_2_-NT/SnO_2_/Ru electrodes before and after electrolysis.

Furthermore, the surface morphology and chemical valence states of the composite on the GF/PDA/TiO_2_-NT/SnO_2_/Ru electrode after electrolysis were investigated. The homogeneous honeycomb structures disappeared after eight cycles of electrolysis (Figure S5a). Instead, more uniform and compact SnO_2_ nanoparticles with an average size of 73.83 nm were formed. The new catalyst structure morphology reduced the permeability of the electrolyte to the SnO_2_ film and prevented anode corrosion, thus prolonging the service lifetime of the electrode. However, large and sharp micron particles of SnO_2_ with an average size of 333.2 nm formed on the GF/PDA/TiO_2_-NT/SnO_2_ electrode (Figure S5b), which was not conducive to the electrocatalytic oxidation. No tin or ruthenium chlorides formed in this process, based on the results of the XRD and XPS (Figure S6–S7). The XPS spectra in Figure 8a indicated that the proportion of Ru^4+^ in the used GF/PDA/TiO_2_-NT/SnO_2_/Ru significantly increased while the proportion of Ru^0^ decreased. In contrast, the content of Sn^2+^ and Ti^3+^ increased by 8.26% and 11.43%, respectively, and the ratio of Sn^4+^ and Ti^4+^ decreased (Figure 8b,c). It can be inferred that Sn and Ti act as active oxidation sites for glyphosate degradation, and Ru acts as the reduction site. Due to the strong coupling interface, Ru could neutralize the excess electrons on SnO_2_ and TiO_2_ to maintain the structural stability of SnO_2_ and TiO_2_. From XPS spectra of O 1s (Figure 8d), for GF/PDA/TiO_2_-NT/SnO_2_/Ru, the intensity of the oxygen vacancy peak increased significantly as the lattice oxygen peak decreased after electrolysis. Therefore, we speculate that the catalytic process of glyphosate on the SnO_2_ and TiO_2_ mainly involves the participation of lattice oxygen in forming active oxygen species such as •O_2_^−^ and the creation of oxygen vacancies.

**Figure 9:**
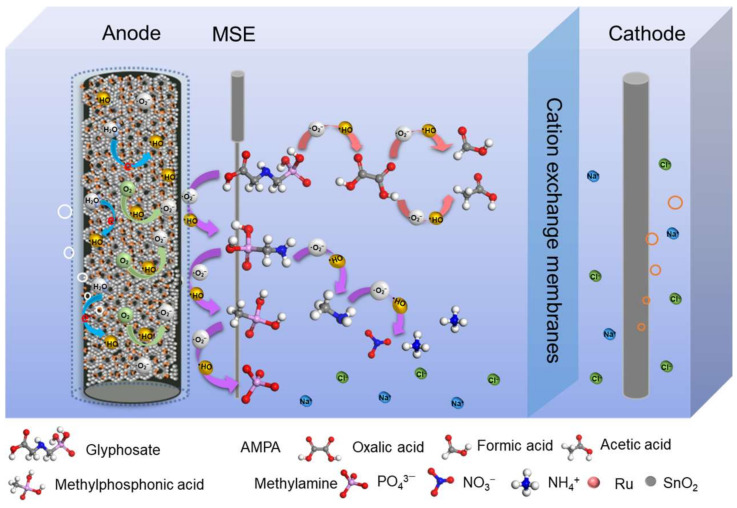
Schematic diagram of the glyphosate degradation on GF/PDA/TiO_2_-NT/SnO_2_/Ru anode in electrocatalytic oxidation process.

The possible degradation pathways of glyphosate on the GF/PDA/TiO_2_-NT/SnO_2_/Ru anode are illustrated in Figure 9. •OH may be formed on the electrode and then reacted with dissolved oxygen to generate •O_2_^−^. The amino group in glyphosate was targeted by •O_2_^−^ radicals, leading to the oxidative cleavage of the C–N bond and the formation of aminomethylphosphonic acid and oxalic acid. Subsequently, aminomethylphosphonic acid was oxidized to produce methylphosphonic acid and methylamine intermediates. Ultimately, the cleavage of the C-P bond in methylphosphonic acid resulted in the formation of PO_4_^3−^. Methylamine was oxidized to N-containing inorganic compounds, such as NH_4_^+^ and NO_3_^−^. Meanwhile, oxalic acid was further oxidized to formic acid and acetic acid intermediates, some of which may be subsequently oxidized to H_2_O and CO_2_.

## 4. Conclusions

A novel GF/PDA/TiO_2_-NT/SnO_2_/Ru anode was prepared by a simple hydrothermal method for efficient electrocatalytic oxidation of glyphosate wastewater. The conversion activity of glyphosate under different process parameters and electrode compositions was investigated. The GF/PDA/TiO_2_-NT/SnO_2_/Ru anode exhibited higher degradation efficiency and stability toward glyphosate than that of the electrodes in the absence of the PDA, TiO_2_-NT, SnO_2_, and Ru. The excellent activity of GF/PDA/TiO_2_-NT/SnO_2_/Ru attributed to its superhydrophilicity improved mass transfer efficiency, and the lattice oxygen in SnO_2_ and TiO_2_-NT generating oxygen to •O_2_^−^ radicals and creation of oxygen vacancies to promote electron transfer. LC-MS and UV spectrophotometry confirmed that the final products of glyphosate were oxalic acid, acetic acid, formic acid, PO_4_^3−^, NH_4_^+^ and NO_3_^−^. This work confirmed that Ru doping activated the lattice oxygen of SnO_2_ and TiO_2_-NT, thus enhancing the degradation efficiency of glyphosate.

## Figures and Tables

**Table 1:** Comparison of the electrocatalytic capacity between GF/PDA/TiO_2_-NT/SnO_2_/Ru and other catalysts toward glyphosate degradation.

Electrodes	Operating Conditions	Removal Efficiency	Energy Consumption	Ref.
BDD	0.59 mM, 10 mA/cm^2^; 0.5 M Na_2_CO_3_, Na_2_SO_4_, NaCl; pH = 3	79% (180 min)	—	[74]
TiO_2_/BDD	0.295 mM, 5 mA/cm^2^; 0.05 M NaCl; pH = 3	91.1% (300 min)	1.39 (Wh/L)	[6]
Ti/Ir_0.3_Sn_0.7_O_2_	5.9 mM, 50 mA/cm^2^; 0.5 M NaCl; pH = 3	91% (240 min)	—	[75]
Ti/Ru_0.36_Ti_0.64_O_2_	0.59 mM, 10 mA/cm^2^; 0.15 M NaCl; pH = 3	>90% (180 min)	10.25 (Wh/L)	[7]
Ti/RuO_2_	0.1 mM, 10 mA/cm^2^; 0.1 M Na_2_SO_4_; pH = 3	80.4% (120 min)	—	[76]
Ti/PbO_2_	0.094 mM, 43 mA/cm^2^; 10 mM Na_2_SO_4_;	95% (360 min)	18 (Wh/L)	[5]
GF/PDA/TiO_2_-NT/SnO_2_/Ru	0.59 mM, 7 mA/cm^2^; 0.5 M NaCl; pH = 3	near to 100% (30 min)	0.088 (Wh/L)	this study

## Data Availability

The data presented in this study are available on request from the corresponding author.

## Supplementary Materials

The following supporting information can be downloaded at: https://www.mdpi.com/article/10.3390/nano14221824/s1, Figure S1: Degradation efficiency of glyphosate on GF, GF/TiO_2_-NT and GF/TiO_2_-NT/SnO_2_/Ru electrodes. Figure S2: LC-MS spectroscopy of glyphosate electrolysis solution at different times (a: 0 min, b,c,d: 5 min, e: 40 min). Figure S3: (a) Influences of glyphosine, NaH_2_PO_3_•5H_2_O, coexistence of glyphosine and NaH_2_PO_3_•5H_2_O on glyphosate degradation; (b) Effect of current densities on glyphosate degradation efficiency in simulated actual glyphosate wastewater. Figure S4. LSV curves of GF/PDA/TiO_2_-NT, GF/PDA/TiO_2_-NT/Ru, GF/PDA/TiO_2_-NT/SnO_2_, GF/PDA/TiO_2_-NT/SnO_2_/Ru electrodes in 0.5 M Na_2_SO_4_, scan rate: 50 mV/s. Figure S5: SEM images of the (a) GF/PDA/TiO_2_-NT/SnO_2_/Ru and (b) GF/PDA/TiO_2_-NT/SnO_2_ electrodes after eight times electrolysis. EDS mapping of the GF/PDA/TiO_2_-NT/SnO_2_ /Ru after eight cycles of electrolysis. Figure S6. Comparison of XRD patterns of GF/PDA/TiO_2_-NT/SnO_2_/Ru electrode before and after degradation of glyphosate. Figure S7. A full-scale XPS spectrum of GF/PDA/TiO_2_-NT/SnO_2_/Ru after degradation of glyphosate. Table S1. Degradation efficiency and energy consumption under different current densities.
