# Supplementary material for: Co-Activating Lattice Oxygen of TiO2-NT and SnO2 Nanoparticles on Superhydrophilic Graphite Felt for Boosting Electrocatalytic Oxidation of Glyphosate
Source: Nanomaterials (Basel). 2024 Nov 14;14(22):1824. doi: 10.3390/nano14221824 (PMC11597287; doi:10.3390/nano14221824)
Supplement: Supplementary file 1 [file nanomaterials-14-01824-s001.zip › nanomaterials-3261931-supplementary.pdf]

Supplementary materials

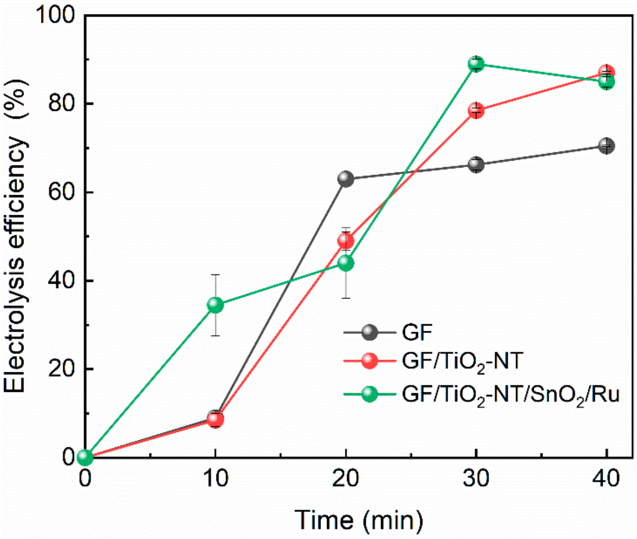

Figure S1. Degradation efficiency of glyphosate on GF, GF/TiO_2-NT and GF/TiO_2-NT/SnO_2/Ru electrodes.

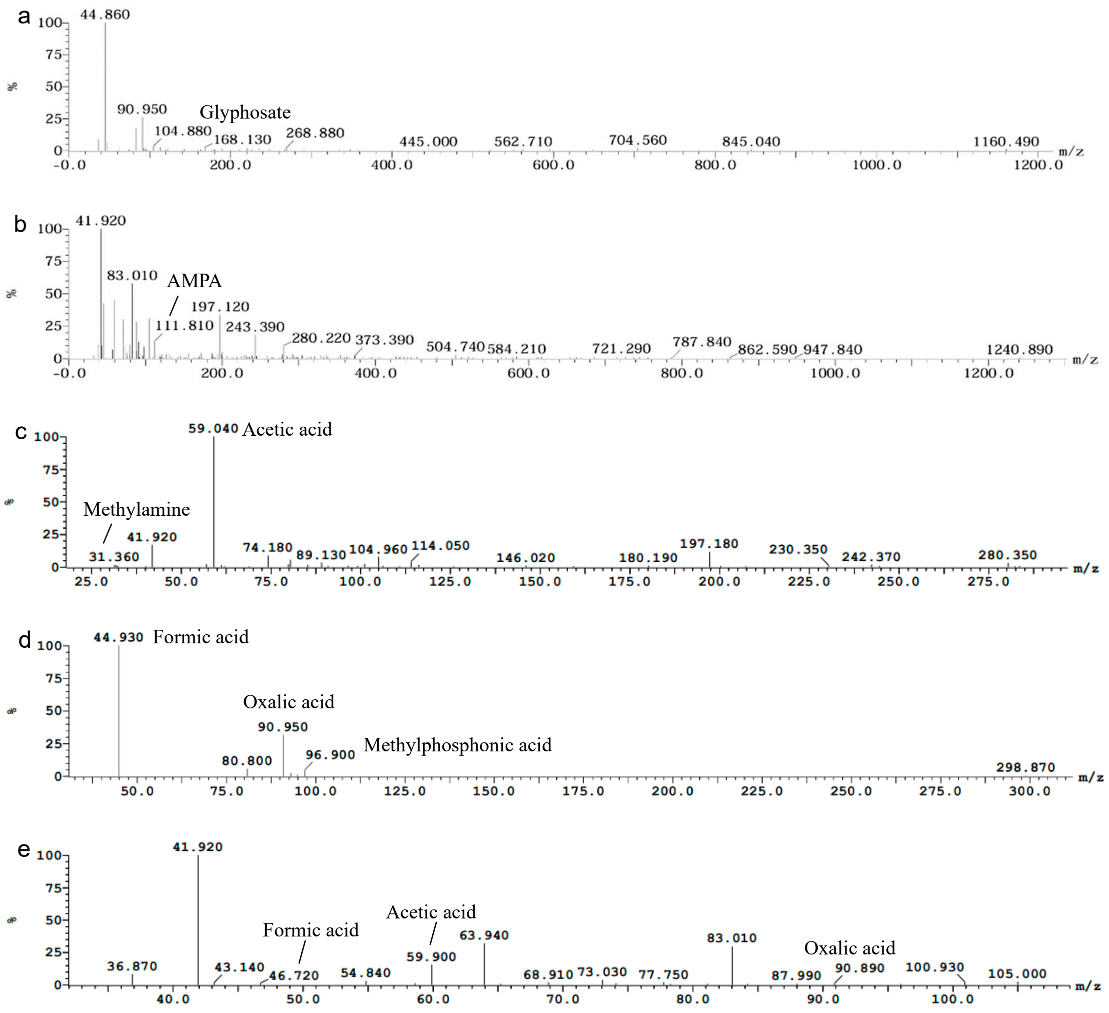

Figure S2. LC-MS spectroscopy of glyphosate electrolysis solution at different times (a: 0 min, b,c,d: 5 min, e: 40 min).

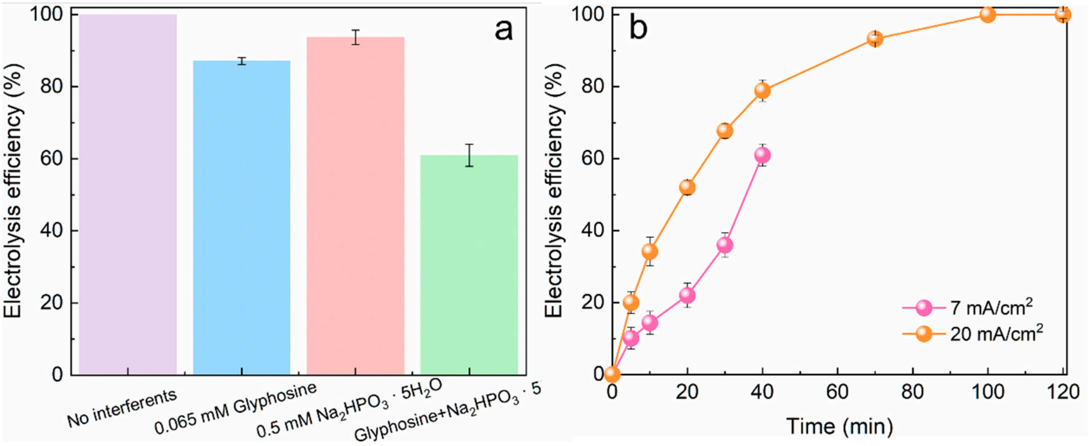

Figure S3. (a) Influences of glyphosine, $NaH_2PO_3 \bullet 5H_2O$, coexistence of glyphosine and $NaH_2PO_3 \bullet 5H_2O$ on glyphosate degradation; (b) Effect of current densities on glyphosate degradation efficiency in simulated actual glyphosate wastewater.

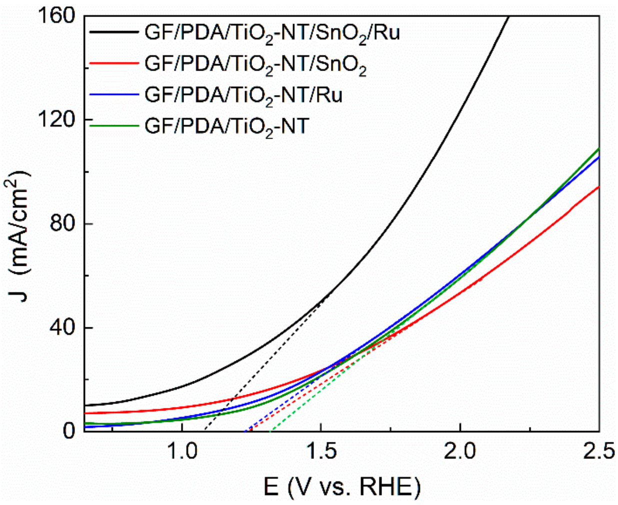

Figure S4. LSV curves of GF/PDA/TiO_2-NT, GF/PDA/TiO_2-NT/Ru, GF/PDA/TiO_2-NT/SnO_2, GF/PDA/TiO_2-NT/SnO_2/Ru electrodes in 0.5 M Na_2SO_4, scan rate: 50 mV/s.

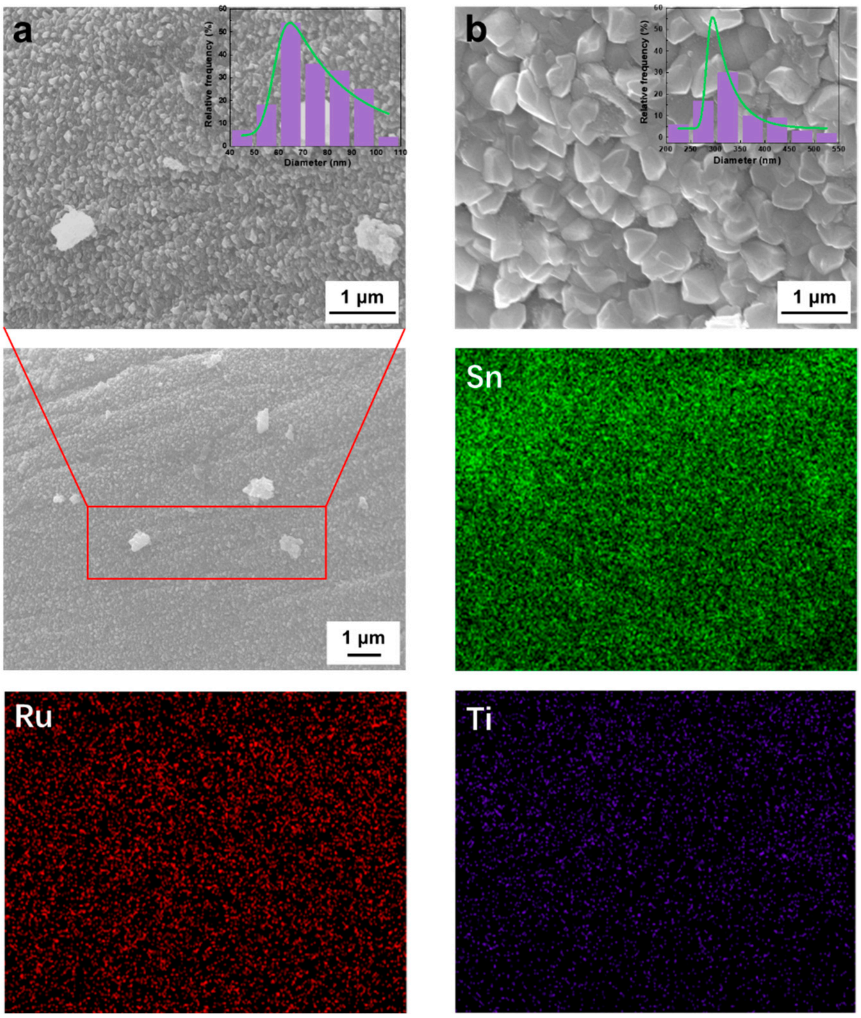

Figure S5. SEM images of the (a) GF/PDA/TiO_2-NT/SnO_2/Ru and (b) GF/PDA/TiO_2-NT/SnO_2 electrodes after eight times electrolysis. EDS mapping of the GF/PDA/TiO_2-NT/SnO_2 /Ru after eight times electrolysis.

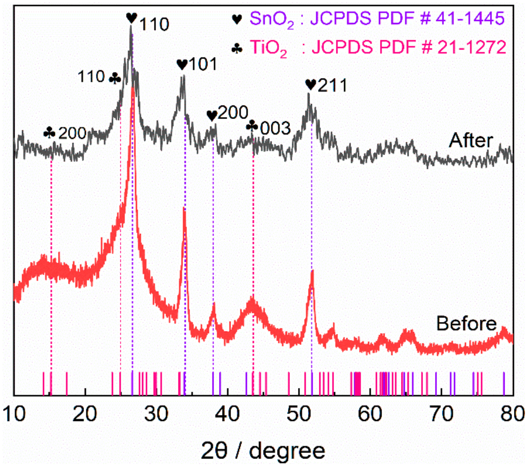

Figure S6. Comparison of XRD patterns of GF/PDA/TiO_2-NT/SnO_2/Ru electrode before and after degradation of glyphosate.

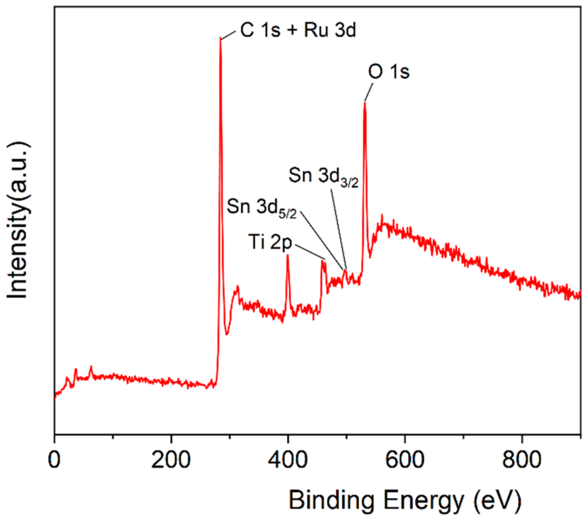

Figure S7. A full-scale XPS spectrum of GF/PDA/TiO_2-NT/SnO_2/Ru after degradation of glyphosate.

Table S1. Degradation efficiency and energy consumption under different current densities.

Current density	Degradation efficiency	Energy consumption
3 mA/cm^2	75%	0.22 Wh/L
5 mA/cm^2	91%	0.21 Wh/L
7 mA/cm^2	100%	0.14 Wh/L
10 mA/cm^2	97%	0.17 Wh/L
